# Exploring Receipt of HIV PEP Counseling Among Women Sexually Assaulted by an Intimate Partner

**DOI:** 10.2174/1874613601812010001

**Published:** 2018-01-31

**Authors:** Janice Du Mont, Lily Van, Daisy Kosa, Sheila Macdonald

**Affiliations:** 1 Women’s College Research Institute, Women’s College Hospital, Toronto, Ontario, Canada; 2 Dalla Lana School of Public Health, University of Toronto, Toronto, Ontario, Canada; 3 Ontario Network of Sexual Assault/Domestic Violence Treatment Centres, Toronto, Ontario, Canada

**Keywords:** Sexual assault, Intimate partner, HIV, Post-exposure prophylaxis, Counseling, Violence

## Abstract

Among 136 women sexually assaulted by a current or former male intimate partner presenting to hospital-based violence treatment centers, 58 (42.6%) received HIV post-exposure prophylaxis (HIV PEP) counseling by a specially trained sexual assault nurse. We identified factors that were associated with receipt of HIV PEP counseling. Those who received counseling were more likely to have been younger than 25 years of age, single, a student, vaginally penetrated, and have received various other services (*e.g*., STI prophylaxis). They were less likely to have been unemployed. Hospital-based violence treatment centers need to be aware that not all women sexually assaulted by an intimate partner will have the same risk of acquisition of HIV and care needs.

## 1 DEAR EDITOR

The World Health Organization recommends counseling to discuss HIV risk and, where appropriate, the risk and benefits of HIV post exposure prophylaxis (PEP) medications as part of comprehensive care for victims of sexual assault [1]. Yet, in our recent study, it was found that receipt of HIV PEP counseling varied substantially by the relationship of the victim to the assailant: women sexually assaulted by an intimate partner were less likely than those assaulted by another known assailant or a stranger to receive HIV PEP counseling (41.0% *vs* 70.6% & 65.8%, respectively; *p *< 0.001) [2]. It is unclear why receipt of HIV PEP counseling was lower among women sexually assaulted by an intimate partner. To shed further light on this issue, we further explored the receipt of HIV PEP counseling among women sexually assaulted by a current or former intimate partner presenting to specialized hospital-based treatment centers.

We examined data prospectively collected from clients who presented to one of 30 participating Sexual Assault/Domestic Violence Treatment Centres (SA/DVTCs) from April 1, 2009 to June 30, 2011 in a larger study of clients use of and satisfaction with services (see [3] for detailed description of the Client Evaluation Project methods). Specialized nurses at the SA/DVTCs offered and provided acute care services to women, men, and children who were victims of sexual assault by any perpetrator, or physical assault by an intimate partner and who presented to a hospital emergency department within approximately 72 hours of assault. Services provided included acute health care, forensic evaluation, risk assessment, safety planning, referral to on-site follow-up care, and referral for ongoing counseling and other supports. Acute health care included crisis counseling, medical care/treatment, emergency contraception, HIV risk counseling, HIV PEP counseling, and testing and prophylactic treatment for sexually transmitted infections including HIV, gonorrhea, chlamydia, and hepatitis B.

Clients who presented to SA/DVTCs were universally and promptly assessed and counseled by the attending nurse for their risk of acquisition of HIV [4]. Those assessed to be at high or unknown risk based on reported or suspected high-risk exposure (*i.e*., vaginal, anal, or oral penetration with or without a condom) and assault by an assailant(s) at unknown or high risk of HIV or who was known to be HIV-positive were immediately offered HIV PEP counseling [5, 6]. This counseling included education regarding indications, benefits, risks, and possible side effects of HIV PEP. Following receipt of counseling, clients who accepted treatment were immediately offered HIV PEP by the attending nurse [5, 6]. These services, including medications, are government-funded (Ontario Ministry of Health and Long-Term Care); the client incurs no direct financial costs.

In the Client Evaluation Project, information recorded by the attending nurse during the visit was based on variables examined and found important in previous sexual assault studies [7-11], and in particular from those which highlighted sexual assault by an intimate partner [12-14]. This information included sociodemographic, assailant, assault, and service receipt characteristics. Ethics approval for this project was attained at all 30 participating hospital-based SA/DVTCs.

The sample analysed in this study included only those women aged 12 years of age and older who were sexually assaulted by a current or former intimate partner (husband/boyfriend). We examined receipt of HIV PEP counseling by sociodemographic, assault, and service receipt characteristics using cross tabulation with Chi-square/Fisher’s exact test with Statistical Package for the Social Sciences (SPSS), version 22.

We found that of 136 women sexually assaulted by a male intimate partner, 58 (42.6%) received HIV PEP counseling. Compared to those who did not receive counseling, these women were more likely to have been younger than 25 years of age (61.4% vs 36.4%, p=0.007), single (71.9% vs 45.5%, p=0.009), and a student (44.6% vs 25.6%, p=0.038). Women who received HIV PEP counseling were less likely to have been unemployed (26.8% vs 47.3%, p=0.023). Furthermore, women who received HIV PEP counseling were more likely than those who did not to have been vaginally penetrated (84.5% vs 62.8%, p=0.010). Receipt of several different types of services was also associated with receipt of HIV PEP counseling including: completion of a Sexual Assault Evidence Kit (81.0% vs 39.7%, p<0.001), anal examination (34.5% vs 16.7%, p=0.028), vaginal examination (69.0% vs 28.6%, p<0.001), prophylaxis for sexually transmitted infections (74.1% vs 35.9%, p<0.001), prophylaxis for pregnancy (62.1% vs 22.8%, p<0.001), and referral to on-site follow-up care (86.2% vs 67.9%, p=0.024) (Table **1**).

This study has several limitations that are important to acknowledge. Although HIV PEP counseling information was collected, the Client Evaluation Project’s primary focus was not HIV and, therefore, no other data about HIV transmission risk were systemically collected (*e.g*., HIV status of the assailant, use of condoms). Furthermore, as receipt of HIV PEP medications was captured as part of receipt of STI prophylaxis as a whole, it is not possible to identity the number of clients who actually accepted treatment with HIV PEP. In addition, there were some data that could have been of value to collect but were not considered feasible to gather during an acute assessment (*e.g*., detailed level of education and income). Finally, data were only collected on whether or not services were received by the client, not whether relevant services were offered and subsequently declined by the client. Therefore, the rate of receipt of HIV PEP counseling in our study may not include all women sexually assaulted by an intimate partner assessed at risk of HIV acquisition. Some women may have declined to engage in HIV PEP counseling due to a lack of concern that their partner had HIV or perhaps reluctance to entertain the possibility that there may have been infidelity in the relationship [15].

Nonetheless, the results of this study may help elucidate why receipt of HIV PEP counseling may have been, in our earlier research, relatively lower among women sexually assaulted by an intimate partner as compared to women assaulted by other assailants [2]. We found that among women sexually assaulted by an intimate partner those who did not receive HIV PEP counseling were more likely to have been older and married, common-law, or co-habiting, and less likely to have identified as a student, a sociodemographic profile that suggests they may be in more established relationships that place them at decreased risk for acquiring HIV. However, women who did not receive counseling were also more likely to be unemployed, often a marker for socioeconomic disadvantage which has been demonstrated to be a barrier to accepting needed health services [16]. Finally, as might be expected, those who did not receive counseling experienced lower rates of penile-vaginal penetration, which may also explain their lower rates of receipt of some health services such as vaginal examination and prophylaxis for sexually transmitted infections and pregnancy.

Findings from our exploratory study suggest that hospital-based violence treatment centers need to be aware that not all women sexually assaulted by current or former intimate partner will have the same risk of acquisition of HIV and care needs. Larger studies are needed to examine the acceptance and receipt of HIV PEP counseling among women sexually assaulted by an intimate partner.

## Figures and Tables

**Table 1:** Sociodemographic, assault, and service use characteristics of women sexually assaulted by a current or former intimate partner, by receipt of HIV PEP counseling (n=136).

**Variable**	**HIV PEP Counseling,** **n = 58** **n (%)**	**No HIV PEP Counseling,** **n = 78** **n (%)**	**P-value**
Sociodemographic Characteristics			
Age			0.007
12 – 24	35 (61.4)	28 (36.4)	
≥ 25	22 (38.6)	49 (63.6)	
Marital Status			0.009
Single	41 (71.9)	35 (45.5)	
Separated, divorced, or widowed	6 (10.5)	15 (19.5)	
Married, common-law, or co-habiting	10 (17.5)	27 (35.1)	
Disability	11 (19.0)	15 (19.2)	1.000
Unemployed	15 (26.8)	35 (47.3)	0.023
Student	25 (44.6)	19 (25.7)	0.038
Assault Characteristics			
Physical Injuries	23 (40.4)	41 (58.6)	0.062
Vaginal Penetration with Penis	49 (84.5)	49 (62.8)	0.010
Anal Penetration with Penis	12 (20.7)	12 (15.4)	0.565
Service Use			
Sexual Assault Evidence Kit Completion	47 (81.0)	31 (39.7)	<0.001
Assessment/Documentation of Injuries	54 (93.1)	66 (84.6)	0.211
Anal Examination	20 (34.5)	13 (16.7)	0.028
Vaginal Examination with Speculum	40 (69.0)	22 (28.6)	<0.001
Medical Care or Treatment	49 (84.5)	54 (69.2)	0.064
Prophylaxis for Sexually Transmitted Infections	43 (74.1)	28 (35.9)	<0.001
Prophylaxis for Pregnancy	36 (62.1)	17 (22.8)	<0.001
Crisis Counseling	41 (70.7)	51 (65.4)	0.639
Risk Assessment	36 (62.1)	38 (48.7)	0.170
Safety Planning	38 (65.5)	53 (67.9)	0.909
Referral to On-Site Follow-Up Care	50 (86.2)	53 (67.9)	0.024

## LIST OF ABBREVIATIONS

(SA/DVTC): Sexual Assault/Domestic Violence Treatment Centre.
(PEP): Post exposure prophylaxis.

## AVAILABILITY OF DATA AND MATERIALS

The data supporting these findings will not be made available publically due to concerns about protecting participants’ identity and respecting their rights to privacy. At the time the study was conducted, we did not obtain informed consent from participants for publication of the dataset.
